# Neuroprotective Potential of Flavonoids in Brain Disorders

**DOI:** 10.3390/brainsci13091258

**Published:** 2023-08-29

**Authors:** Syed Hasan, Nabeel Khatri, Zainab N. Rahman, Amanda A. Menezes, Joud Martini, Faheem Shehjar, Numa Mujeeb, Zahoor A. Shah

**Affiliations:** 1 Department of Pharmacology and Experimental Therapeutics, College of Pharmacy and Pharmaceutical Sciences, The University of Toledo, 3000 Arlington Avenue, Toledo, OH 43614, USA; 2 Department of Medicinal and Biological Chemistry, The University of Toledo, 3000 Arlington Avenue, Toledo, OH 43614, USA

**Keywords:** flavonoids, neuroprotection, neuroinflammation, brain injury, stroke

## Abstract

Flavonoids are a large subgroup of polyphenols known to be sourced from over 6000 natural products, including fruits, vegetables, bark, and herbs. Due to their antioxidant properties, flavonoids have been implicated as a therapy source for many diseases and conditions, including inflammation, vasculitis, venous insufficiency, and hemorrhoids. Currently, some flavonoids are being researched for their antioxidant ability concerning neuroprotection. These flavonoids can penetrate the blood–brain barrier and, depending on the specific flavonoid, retain adequate bioavailability in certain brain regions. Further data suggest that flavonoids could have a strong anti-inflammatory effect in the brain, which not only could be a robust therapeutic source for known neuroinflammatory diseases such as Alzheimer’s Disease or Parkinson’s Disease but also could be a therapeutic source for ischemic or hemorrhagic conditions such as a stroke. While flavonoid toxicity exists, they are relatively safe and non-invasive drugs from natural origins. As such, exploring the known mechanisms and therapies may highlight and establish flavonoid therapy as a viable source of therapy for stroke patients. As stated, many flavonoids are already being isolated, purified, and implemented in both in vitro and in vivo experiments. As these flavonoids proceed to clinical trials, it will be important to understand how they function as a therapy, primarily as antioxidants, and by other secondary mechanisms. This review aims to elucidate those mechanisms and explore the neuroprotective role of flavonoids.

## 1. Introduction

Very few physical injuries are as intimidating and life-threatening as a head or brain injury. According to epidemiological studies, roughly 5% of Americans suffer from traumatic brain injuries (TBIs) [1]. An acquired brain injury can result from a physical blow, such as a car accident or sports injury; it can also occur because of ischemic or hemorrhagic conditions; both states substantially affect brain health. While the outcomes are relatively the same for both situations, the main difference between them is the type of injury to the brain [1,2,3]. While both conditions are forms of what is called “Acquired Brain Injury”, they both fall into separate categories of traumatic brain injury (TBI) and non-traumatic brain injury (nTBI) due to the type of damage. External factors, such as physical injury, come from outside our body and result in TBI. In contrast, the internal factors that come from inside of our body and affect our brain vasculature, metabolism, and plasticity can result in strokes, seizures, tumors, infections, and/or hypoxia/anoxia; these are considered forms of nTBI [4,5,6].

TBI can further be divided into sub-categories that are caused by external factors. These sub-categories of TBI are penetrating and non-penetrating brain injury [7,8]. Penetrating brain injury is defined as a wound to the cranium in which a projectile breaches the cranium and does not exist. Non-penetrating can be defined as blunt trauma or force to the cranium and are the more common form of traumatic brain injury [7,9]. While penetrating brain injuries are fatal and challenging to treat, both types of injuries are quite dangerous. They currently affect roughly 5.3 million Americans, which is approximately 2 percent of the population. Furthermore, as reported by the Center for Disease Control and Prevention (CDC), it is the leading cause of disability and death in children and adolescents in the United States.

Symptoms of traumatic brain injury can be broken down into three categories: physical, behavioral, and sensational [10]. Physical symptoms can include headache, nausea, and dilated pupils. Behavioral symptoms include sleeping disorders, lack of awareness or attention, and pronounced anxiolytic irritability. Sensational symptoms of TBI are dizziness, fatigue, altered visual/auditory function, and vestibular dysfunction. 

The other form of brain injury, as discussed, is non-traumatic brain injury (nTBI). nTBI is usually the result of internal factors such as an occluded brain vasculature, hypoxia, strokes, and neurodegenerative diseases like Alzheimer’s Disease or Parkinson’s Disease [11].

One of the most important “internal factors” that causes “Acquired Brain Injury” (ABI) is stroke. According to the CDC, strokes are defined as a brain attack when there is occlusion or damage to the blood vessels in the brain. This can lead to permanent damage to the brain in the form of nutrient deprivation, oxygen deprivation, and an increased risk of infection [12]. The CDC reports that approximately 800,000 people a year have a stroke, 87% of which are ischemic. Ischemic strokes are defined as a loss of blood supply to a region or area of the brain, in contrast to hemorrhagic strokes, which are defined as an active bleed occurring due to a rupture in the blood vessels in the brain [13]. Strokes can be very detrimental to the point of fatality in humans; the CDC reports that statistically, every 3 min, someone in the United States dies of a stroke or complications from one [12].

Strokes are one of many diseases affecting the brain, leading to rapid cell death. Due to the occlusion of blood vessels in the form of a blood clot or a buildup of fatty acid plaques [14], the brain will not receive enough oxygen and nutrients to function properly and retain plasticity. Hemorrhagic strokes can also occur due to aneurysms categorized by a dramatic increase in intracranial pressure, leading to drastic and potentially permanent damage to the brain tissue.

According to the “American Heart Association,” changing lifestyle habits will help to prevent 80% of stroke chances: for example, monitoring blood pressure regularly, avoiding smoking and drinking alcohol, and following a healthy diet. Diet is a significantly strong risk factor for stroke patients. A healthy diet has been shown to reduce the risk of strokes by over 80% [15]. 

**Figure 1:**
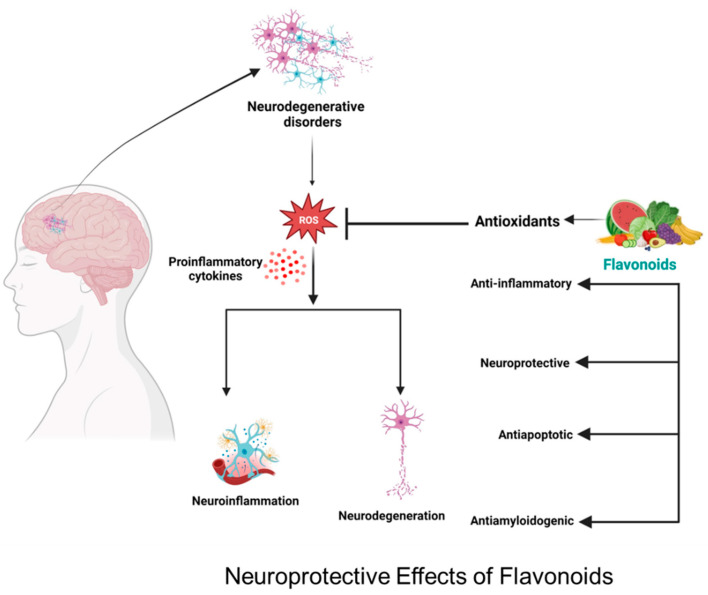
Neuroprotective effect of flavonoids in neuroinflammation and neurodegeneration through different mechanisms of action.

A healthy diet must include plant sources such as fruits, vegetables, and grains. Flavonoids are chemical substances found primarily in fruit and vegetables and form a rich source of a healthy diet. Flavonoids are antioxidants that help reduce oxidative stress in our bodies, such as free radicals [16]. Furthermore, they work as anti-inflammatory agents that can help reduce the negative symptoms of inflammation. Indeed, it is apparent that flavonoids could benefit stroke patients; this review will discuss various flavonoids, the mechanism of their action in the brain, and their potential role in treating brain injury (Figure 1). 

## 2. Flavonoids

**Figure 2:**
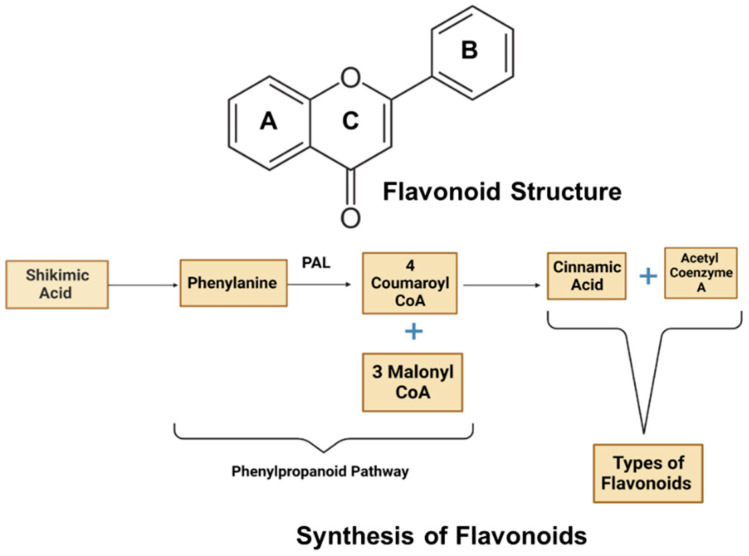
General chemical structure and synthesis of flavonoids. The general chemical structure of flavonoids consists of a 15-carbon flavone skeleton, C6-C3-C6, with two pyran benzene rings (A and B) linked by a three-carbon heterocyclic oxygen-containing pyran ring C.

Flavonoids, belonging to a class of secondary metabolites, are polyphenolic compounds. The general chemical structure of flavonoids consists of a 15-carbon flavone skeleton (Figure 1), C6-C3-C6, with two pyran benzene rings (A and B) linked by a three-carbon heterocyclic oxygen-containing pyran ring C [17]. The positioning of the phenyl ring and hydroxyl group directs flavonoids’ antioxidative capacity and classification into various subgroups [18]. Many naturally occurring sources of flavonoids have been isolated and purified. Flavonoids are synthesized via the phenylpropanoid pathway in which phenylalanine is converted into 4-coumaroyl-CoA, which finally enters the flavonoid biosynthesis pathway (Figure 2). The combination of shikimate and acetate enzymatically releases several moieties, with chalcones becoming the intermediates for the biosynthesis of flavonoid classes [19]. 

### 2.1. Mechanisms of Flavonoid Therapy

In rat models, several flavonoids have been found to prevent AD from developing and to cure cognitive deficits, indicating their potential therapeutic value. Previous research has focused on the anti-amyloidogenic activities of flavonoids since altered amyloid precursor protein (APP) processing leads to increased Aβ formation, which is a significant pathogenic characteristic of AD. It has recently been shown that bilberry and black currant extracts rich in anthocyanins have the power to alter APP processing and lessen behavioral impairments in the APP/PS1 mice model of AD [20].

Several studies support the effectiveness of flavonoids on memory and learning. For instance, nobiletin, a citrus flavonoid, improved Aβ-induced memory impairment and reduced Aβ load and plaques in the hippocampus of a transgenic mice model of AD [21]. Additionally, *Tg2576* mice given grape-derived polyphenols (GSPEs) orally for five months show less cognitive decline and lower levels of high-molecular-weight soluble Aβ oligomers in the brain [22]. In human “Swedish” mutant APP transgene-bearing neuron-like cells and primary neurons, luteolin, another citrus flavonoid, was found to decrease Aβ peptide production and amyloidogenic gamma-secretase APP processing [23]. Additionally, a 9-month regimen of curcumin or grape seed extract high in polyphenols stopped the buildup of amyloid-beta in an AD animal model [24]. In a transgenic mouse model of AD, it was shown that long-term (16-month) treatment with Ginkgo biloba extract (EGb761) significantly reduced APP protein levels, indicating that its potential neuroprotective properties may be, at least in part, related to its APP lowering effects [25]. 

Tannic acid treatment in PSAPP (*Presenilin-1* and amyloid precursor protein overpression) mice also decreased brain parenchymal and cerebral vascular β-amyloid deposits, indicating that it functions as a natural β-secretase inhibitor [26]. In primary cortical neurons, natural flavonoids have been demonstrated to potently inhibit BACE-1 activity and decrease the amount of released Aβ [27]. Similarly, curcumin and (−)-epigallocatechin-3-gallate (EGCG) repress amyloid beta-induced BACE-1 overexpression in neuronal cultures [28]. Studies on the advantages of regular green tea use have received much attention; by modifying the production of amyloid precursor protein (APP), green tea polyphenol EGCG helps lower brain Aβ levels [29,30]. For EGCG to promote non-amyloidogenic (α-secretase cleavage) APP processing, ADAM10 activation is required [31]. Additionally, it was discovered that the maturation of ADAM10, via an estrogen receptor-/phosphoinositide 3-kinase-/Ak-transforming-dependent mechanism, mediates the EGCG-mediated improvement of non-amyloidogenic processing of APP. Selective estrogen receptor modulation may be a therapeutic goal due to the correlation between estrogen depletion after menopause and an increased risk of developing AD. EGCG may be an alternative to estrogen therapy in preventing and treating this condition [32]. 

By being able to prevent the development of β-sheet-rich amyloid fibrils, EGCG may also have neuroprotective effects. It was proven to contain the naturally unfolded polypeptides that inhibits Aβ aggregation and fibrillogenesis [33]. Additionally, EGCG can reduce giant amyloid-beta fibrils into smaller, harmless amorphous protein aggregates, indicating that it is an effective remodeling agent for mature amyloid fibrils [34]. Other flavonoids have also demonstrated anti-amyloidogenic activities, particularly myricetin, which inhibits the formation of amyloid beta-plaques in vitro by preferentially and irreversibly attaching to the amyloid fibril structure rather than to Aβ monomers [35,36]. Overall, these results indicate that certain flavonoids can interfere with fibrillization by producing off-target Aβ oligomers, acting as BACE-1 inhibitors, or boosting ADAM10 activity, reducing the synthesis of Aβ. 

### 2.2. Tau and Flavonoids

Flavonoids’ potential anti-AD properties could influence downstream targets like tau phosphorylation. Regarding this, certain features of studies have clarified the role of flavonoids in the tau protein, which may impact AD. EGCG and myricetin have been demonstrated to decrease heparin-induced tau production, and EGCG treatment in Alzheimer transgenic mice modifies tau profiles by suppressing the isoforms of phosphorylated tau that are sarkosyl-soluble [30]. Other research using GSPE has demonstrated its capacity to prevent tau peptide aggregations, dissociate already formed tau peptide aggregates, and disrupt PHFs in a mouse model of AD [22,37,38,39]. 

### 2.3. Anti-Inflammatory Actions of Flavonoids 

High levels of low-density lipoprotein and total cholesterol are known to cause severe oxidative stress and lipid oxidation that causes lipids to build up in the vessel walls, foam cells to form, vascular muscle cells to migrate and multiply, and an inflammatory response to be triggered, all of which lead to cardiovascular damage [40,41,42,43]. C-reactive protein (CRP), cytokines (IL-6, IL-1, TNF-α), reactive oxygen species (ROS), adhesion molecules (ICAM-1, VCAM-1, selectins), and several other molecules are all involved in inflammation [44,45,46,47]. Studies have revealed that flavonoids have various anti-inflammatory effects, primarily via scavenging ROS, inhibiting the activity of certain enzymes like cyclooxygenases and lipoxygenases, activating eNOS, and changing gene expression. Cyclooxygenases and lipoxygenases are required for the metabolism of arachidonic acid and the generation of a variety of metabolites, including prostaglandins, leukotrienes, and thromboxanes, which exacerbate the inflammatory response [48]. Flavonoids effectively inhibit COX-2, which overreacts in inflammation, while only weakly inhibiting COX-1 by decreasing its expression [49,50]: for instance, in rat macrophages, quercetin and kaempferol inhibit COX-2 [51]. Additionally, flavonols (kaemferol, quercetin, morin, and myricetin) are more effective lipoxygenase inhibitors than flavones, which reduces the formation of leukotrienes. Flavonoids also stimulate the activity of antioxidant enzymes such as glutathione reductase, glutathione peroxidase, heme-oxygenase, g-glutamyl cysteine synthetase, and superoxide dismutase [52,53,54]. Additionally, flavonoids decrease NO overproduction by directly scavenging NO, inhibiting iNOS, and inhibiting the synthesis of iNOS protein. The 2 and 3-double bonds in flavanones and adequate lipophilicity are the essential structural components that cause this activity. For instance, higher methyl and alkyl group-containing flavonoids are significantly more active than hydrophilic and glycosylated flavonoids [55,56]. Flavonoids function through the suppression of the NF-kB pathway in addition to directly inhibiting the synthesis of the iNOS protein [49,55,57,58]. Additionally, flavonoids have been shown to upregulate the expression of the anti-inflammatory cytokine IL-10 and reduce interferon-mediated cytotoxicity while downregulating the expression of pro-inflammatory cytokines such as IL-1β, IL-6, IL-8, TNF-α, and monocyte-chemoattractant protein-1 [59,60]. The primary mechanism involves the inhibition of MAPK, binding of AP-1 to DNA, and downregulation of iNOS synthase [61].

### 2.4. Antiplatelet Actions of Flavonoids 

Numerous studies have demonstrated flavonoids’ capacity to prevent the production of thrombi. The presence of the 2, 3-double bonds, the 4-keto group, the 7-hydroxyl group, and the 4-hydroxyl group is required for this activity. Changes to this structure or glycosylation may significantly diminish the antiplatelet effect [62,63].

Several different mechanisms mediate flavonoids’ antiplatelet action. First, flavonoids temporarily block thromboxane A2 receptor activation [62,63]. The antioxidant abilities of flavonoids, which are enhanced by increased eNOS activity, account for a sizable portion of these effects [64,65]. Additionally, flavonoids from red wine and grape extracts stimulate the platelet-endothelial cell adhesion molecule-1 molecule with anticoagulant characteristics [66]. Furthermore, flavonoids have been associated with reduced p-selectin expression and the release of p-selectin microparticles at the beginning of coagulation [67]. Furthermore, it was shown that by inhibiting acetyl-CoA-acetyltransferase, which lowers the level of synthesis, tea catechins had been demonstrated to reduce the generation of the platelet-activating factor [68]. 

### 2.5. Flavonoids and the Blood–Brain Barrier (BBB)

When discussing brain injury and treatment, it is imperative to understand the pharmacokinetics of flavonoids and how they can penetrate the BBB. Indeed, it is well known and well studied that most flavonoids follow the Lipinski rule of 5, which predicts pharmacokinetics, specifically absorption, distribution, metabolism, and excretion (ADME) [69]. As most drugs should follow these guidelines, it is crucial to understand the pharmacokinetic profile of each drug and the predicted carcinogenic and toxicological effects of certain flavonoids. These predictive or molecular docking studies were performed in silico and are very insightful regarding using certain chemicals as drug treatments. Based on these molecular docking studies, it can be determined that flavonoids can indeed be a potential therapy for brain injury in terms of pharmacokinetic properties and molecular docking or targeting. 

The penetration of flavonoids into the BBB has been well known and well defined, dating back to a study in London where researchers specifically looked at the BBB penetration properties of flavonoids [70] in both an in vitro blood–brain model of human brain endothelial cells and brain endothelial cell lines derived from both mice (b.END5) and rats (RBE4). Researchers found that the uptake of flavonoids occurred in both cell lines, indicating that the endothelial BBB will interact positively with various flavonoids.

The uptake of flavonoids from the brain endothelium infers that several flavonoids could readily interact and cross the BBB through therapy and treatment. Furthermore, this interaction has also been shown to occur at the microvasculature level, indicating viable penetration into the BBB, which could lead to downstream therapeutic effects; as b.END5 and RBE4 cells do not accurately form tight junctions, the utilization of a more accurate permeability model was also employed using a well-established in vitro model for BBB interactions. Youdim et al. studied the interaction between two flavonoids and their respective permeabilities across the BBB, utilizing ECV304/C6 cells to more accurately mimic the tight junctions formed by the epithelium in the brain [71].

Furthermore, they also tested the permeability of the two flavonoids, quercetin, and naringenin, which were also studied in an in situ rat model. Their studies show flavonoids can easily cross the BBB and enter different brain regions. However, the degree to which they do can vary between flavonoids. For example, in their studies, Youdim et al. found that naringenin does cross the BBB more readily than quercetin. Furthermore, it can even penetrate as far as the central nervous system. Quercetin, on the other hand, did cross the BBB and was found to interact with p-glycoprotein potentially; researchers hypothesize that the flavonoid acts as a type of substrate for the protein, which has been implied in many different neurodegenerative diseases such as Alzheimer’s Disease, Parkinson’s Disease, and frontotemporal dementia [72]. Overall, it is evident that flavonoids, as a general chemical class, can cross the BBB readily and, as such, are widely used in treating different diseases that affect the brain. 

## 3. Flavonoid Neuropharmacology

As stated, it has been determined for some time that certain flavonoids can cross the BBB; here, we will discuss the current treatments used for various brain diseases. 

### 3.1. Neuroinflammation

Two major hallmark diseases centered around neuroinflammation are Alzheimer’s Disease (AD) and Parkinson’s Disease (PD) [73]. Neuroinflammation has been known to be a critical factor in these diseases; understanding the role of anti-inflammatory agents, such as flavonoids, could be beneficial in developing future treatments. Recently, a clinical trial was performed for a therapeutic form of genistein, a well-defined flavonoid chemical, on prodromal Alzheimer’s patients [74]. It was found that placebo-treated patients scored lower on cognitive tests and showed further AD progression. They concluded that genistein could significantly impede the progression of AD and that the treatment group showed exactly those results in cognition tests and 18F-flutemetamol PET scan for amyloid-beta deposition. The trial coined the GENIAL trial implies that even with the emergence of new therapies, such as biological or antibody therapies, flavonoids still could present a practical and relatively safe avenue of treatment for specific individuals. 

Another study showed a simple fruit juice diet in middle-aged women with worsening cognition improved their cognitive performances on the Rey Auditory Verbal Learning Test [75]. This study further associated the presence of polyphenols in the blood and urine, which are known metabolites of fruit juice. Urine analysis in this study revealed higher levels of thyroxine and 3-methyladenine, which yield stability to pro-autophagic signaling associated with AD. Further clinical trials will elucidate additional mechanistic and therapeutic information regarding genistein and polyphenols and their role in neuroinflammatory diseases.

One study involved blackcurrant anthocyanins as a dietary supplement, and consequent cerebrospinal fluid analysis was performed [76] to look for insulin-like growth factor 1 (IGF-1), insulin-like growth factor binding protein 3 (IGFBP-3), and cyclic glycine-proline (cGP), which is a known anthocyanin metabolite with neuroprotective properties. They found increased levels of cGP but not IGF-1 or IGFBP-3. This led the researchers to conclude that blackcurrant anthocyanins could help stabilize IGF-1 through the neuroprotective properties of cGP, indicating that blackcurrant anthocyanins could help mitigate the progression of diseases such as PD, where IGF-1 has been known to be underregulated.

### 3.2. Brain Cancer

A study showed the effects of several flavonoids on human GL-15 glioblastoma cells and found that all the flavonoids decreased the number of viable cells for cancer proliferation [77]. The anti-cancer effects shown by flavonoids indicate that flavonoids could have cytotoxic effects on cancer cells and could be a vital avenue of therapy for patients with early forms of brain gliomas. 

Indeed, flavonoids have a role in different brain diseases. As they readily cross the BBB, they are ideal candidates for therapy, given that they are sourced primarily through natural products. While it has been shown that they can have a role in slowing the progression of diseases such as AD, PD, and cancer, here, we will discuss the role of flavonoids and their neuroprotective characteristics, which could help treat stroke patients or other forms of Acquired Brain Injury (ABI). 

### 3.3. Flavonoids and Stroke 

Over the past few decades, many studies have been conducted, unveiling intriguing insights into using flavonoids as a remedy for stroke. These studies have explored the therapeutic potential of flavonoids, delving into their unique properties and mechanisms of action, aiming to harness their beneficial effects in mitigating the impact of stroke. According to a notable study published in *The Lancet* in 2006 [78], evidence supports the association between high polyphenolic content in food and a reduced risk of stroke. They found that individuals who regularly consumed higher amounts of fruits and vegetables, which are rich sources of polyphenols, exhibited a lower risk of experiencing a stroke.

In a compelling randomized trial conducted by Bellone et al. [79], the researchers investigated the potential benefits of pomegranate supplementation in ischemic stroke recovery. The researchers recruited a group of individuals who had experienced an ischemic stroke and randomly assigned them to receive either pomegranate supplementation or a placebo. Cognitive and functional assessments were performed at baseline and after a designated intervention period. The trial results demonstrated that participants who received pomegranate supplementation exhibited significant improvements in cognitive function and functional recovery compared to those in the placebo group. These improvements were particularly evident in domains such as memory, attention, and overall neurological function. The findings suggested that the bioactive compounds present in pomegranate may have played a crucial role in promoting neuroplasticity and recovery following ischemic stroke. Another study by Zhang et al. showed that fisetin, a flavonoid found in pomegranates [80], activates the Nrf2–ARE pathway, which is a key cellular defense mechanism against oxidative damage [81]. The researchers conducted experiments using a TBI-induced rat model and administered fisetin to evaluate its effects on oxidative stress markers and the activation of the Nrf2–ARE pathway. They observed that fisetin treatment significantly attenuated oxidative stress, as evidenced by reduced levels of reactive oxygen species (ROS) and increased levels of antioxidant enzymes. These findings suggest that fisetin’s neuroprotective effects may be mediated, at least in part, by its ability to enhance the antioxidant capacity of brain cells through the Nrf2–ARE pathway. The findings in this study suggest that polyphenols may be responsible for promoting neuroplasticity and recovery following ischemic stroke. The experimenters conducted experiments on a TBI-induced rat model and administered fisetin to assess its effects on oxidative stress markers and the transcriptional Nrf2–ARE pathway. They reported that fisetin treatment significantly attenuated oxidative stress, as shown by reduced levels of reactive oxygen species (ROS) and increased levels of antioxidant enzymes.

One study focused on a large cohort of women and utilized comprehensive dietary assessments, and the study provided valuable insights into the potential protective effects of specific flavonoid subclasses [82]. Flavanones, found predominantly in citrus fruits, exhibited the strongest inverse association with stroke risk, which were followed by anthocyanins, abundant in berries, and flavones, commonly found in parsley and celery. These findings suggest that consuming a diet rich in flavonoids, particularly these subclasses, may reduce stroke risk. Another study provides valuable insights into pinocembrin, which is a flavanone with promising therapeutic potential [83]. Pinocembrin, derived from natural sources like propolis and honey, exhibits a range of pharmacological activities that make it a promising candidate for stroke therapy. The compound demonstrates antioxidant properties, helping to combat oxidative stress, which is a significant contributor to stroke development. 

Cerebral ischemia–reperfusion injury, a critical event in the pathogenesis of stroke, involves complex mechanisms such as apoptosis and extracellular matrix (ECM) accumulation. One study, for example, highlights the significance of apoptotic cell death in mediating the pathological consequences of acute brain ischemia and emphasizes the need for targeted therapeutic interventions to modulate apoptotic pathways to mitigate the detrimental effects of ischemic stroke [84]. This study allows for a target point of research to mitigate the impact of stroke, which was accomplished in a 2021 study by Wu et al. It provides valuable insights into the neuroprotective effects of icaritin and icariin in an experimental model of ischemic stroke. The findings highlight the ability of these flavonoids to mitigate neuronal apoptosis, preserve brain tissue integrity, and reduce the excessive accumulation of ECM following ischemic injury. Wu et al. demonstrated that treatment with icaritin and icariin significantly reduces apoptotic markers, such as caspase-3 and Bax, which are known to play crucial roles in the apoptotic cascade [85].

Conversely, the expression of anti-apoptotic proteins, including Bcl-2, is upregulated in response to flavonoid administration, further enhancing the cell survival mechanisms. In addition to their anti-apoptotic effects, icaritin and icariin exhibit significant inhibitory activity against ECM accumulation. The flavonoids effectively suppress the activity of matrix metalloproteinases (MMPs), which are enzymes responsible for ECM degradation, thereby preventing excessive ECM deposition [86]. Simultaneously, they upregulate the expression of tissue inhibitors of metalloproteinases (TIMPs), which contribute to maintaining the ECM balance and preserving the integrity of brain tissue. The study provides valuable insights into the mechanisms underlying the protective effects of these flavonoids and highlights their translational potential for stroke management. 

Hemorrhagic brain injury or intracranial hemorrhage (ICH), characterized by bleeding into the brain parenchyma or subarachnoid space, threatens neurological function and patient outcomes [13]. Shi et al. focused on baicalin, a specific flavonoid compound and active ingredient of the Chinese herbal medicine *Scutellaria baicalensis*, and its protective effects in a mouse model of subarachnoid hemorrhage [87]. Baicalin treatment was found to attenuate BBB disruption, which is a critical event that exacerbates brain edema and secondary brain damage following hemorrhage. Baicalin exerted its protective effects by modulating the expression of tight junction proteins and reducing vascular permeability, thereby preserving the integrity of the BBB. The flavonoid compound suppressed the activation of pro-inflammatory signaling pathways and inhibited the release of inflammatory mediators, such as IL-1β and TNFα. By attenuating the inflammatory response, baicalin helped to mitigate the harmful effects of excessive immune activation and inflammatory cell infiltration in the brain. The study also revealed that baicalin exhibited potent antioxidant properties. Oxidative stress is highly associated with hemorrhagic brain injury, contributing to neuronal damage and functional impairment [88]. Baicalin treatment effectively reduced reactive oxygen species and enhanced endogenous antioxidant defenses, thereby reducing oxidative damage and promoting neuronal survival in the injured brain. 

ICH is a devastating condition that comprises 10–15% of all strokes and is associated with high morbidity and mortality [89]. While rutin, a dietary flavonoid, has demonstrated neuroprotective effects against cerebral ischemic stroke through its antioxidant and anti-inflammatory properties, its potential efficacy against ICH stroke remains unexplored [90]. In a study by Rana et al. [91], the researchers investigated the impact of rutin in an ICH zebrafish larva model. The results revealed that rutin administration effectively reduced hematoma size, inhibited ROS production, and mitigated apoptosis in the brains of zebrafish larvae with ICH. Additionally, rutin treatment reduced malondialdehyde and protein carbonyl levels, which is indicative of its ability to counteract free radical-induced damage. Rutin treatment upregulated the expression of the tight junction claud5a gene while downregulating the mRNA levels of matrix metalloproteases, MMP2, and MMP9, thus contributing to the preservation of BBB integrity. Furthermore, rutin treatment attenuated the genomic expression of oxidative stress markers and inflammatory genes associated with ICH. The study highlights rutin’s ability to alleviate oxidative stress, inhibit inflammatory processes, and maintain crucial signaling pathways involved in cellular homeostasis. 

This section of the review article explores the potential of flavonoids as therapeutic agents for mitigating the detrimental effects of ICH, drawing insights from the study conducted by [92]. The research investigates the clinical efficacy and computed tomography (CT) perfusion of puerarin combined with naloxone in treating traumatic cerebral infarction, which is a condition related to ICH. A systematic review by Turner et al. conducted in 2021 showed that TBI patients have an 86% increased risk of stroke compared to non-TBI controls [93]. The study by Lubo et al. focuses on puerarin, a flavonoid compound derived from the herb *Pueraria lobata*, and its combination with naloxone in treating traumatic cerebral infarction [92]. The study’s findings demonstrated that the combination treatment of puerarin and naloxone led to improved clinical outcomes, including the resolution of symptoms and reduced complications associated with traumatic cerebral infarction. Further research with a larger sample size is needed to understand and implement this flavonoid’s therapeutic effects in the stroke context.

Another study investigated the potential of long-term resveratrol supplementation as a secondary prophylaxis for stroke [94]. They aimed to evaluate the effects of resveratrol as a secondary prevention strategy for individuals with a history of stroke. The study revealed that long-term resveratrol supplementation improved various risk factors associated with stroke recurrence. Participants receiving resveratrol exhibited significant improvements in blood pressure regulation, lipid profiles, and markers of inflammation compared to the placebo group. Resveratrol also serves as an adjuvant with r-tPA treatment, which extends the clinical therapeutic window of r-tPA, thereby improving the outcome of patients receiving late stroke treatment [95]. By targeting MMPs, resveratrol holds promise as a therapeutic agent for optimizing the outcomes of delayed r-tPA treatment, providing a potential avenue for improving stroke care and patient prognosis.

The impact of dietary flavonoids on stroke prevention remains unclear. Multiple studies have reported the effect of total flavone intake concerning stroke protection, with more recent studies focusing on the flavone subclass. In the Nurses’ Health Study, women who increased their intake of the flavanone sub-class were less likely to experience an ischemic stroke. According to the study, a higher consumption of flavonoid intake was associated with a 19% reduction in the risk of acute ischemic stroke (AIS). However, the reduction was inversely related to higher citrus fruit consumption than the total flavonoid intake [82].

## 4. Toxic Effects of Flavonoids

As the saying goes, “Too much of anything is bad;” flavonoids taken in large amounts can harm your body instead of protecting it. The majority of the studies in the past have demonstrated the beneficial effects of flavonoids in many diseases, including neurodegenerative diseases such as AD, PD, ischemic stroke, TBI, etc. However, it is essential to consider the potential adverse effects and toxicity associated with their consumption. Although flavonoids are considered safe and well-tolerated when obtained from dietary sources, they must always be taken after consulting physicians.

There have been reports about an excessive consumption of flavonoids leading to significant morbidity and mortality in patients [96]. This could be due to the excessive consumption of flavonoids in the supplemental form, especially in more vulnerable populations such as older adults, or due to the detrimental drug interactions, as it is well known that many flavonoids could potentially interact with some prescription medication [97].

Since research primarily investigates the beneficial health effects of flavonoids, toxicity and other side effects are often underreported. One important consideration is the mutagenicity of flavonoids. It has been shown previously that some flavonoid extracts from food or herbal sources have a potential mutagenic effect linked to the concentration of flavones. Therefore, it is of utmost importance to thoroughly study the side effects of flavonoids before declaring them safe for supplemental use [98]. The toxicological profile associated with various flavonoids preparations in the market remains an area of limited research, as most of these flavonoid preparations available as herbal medicines or dietary supplements, with different claimed therapeutic effects, have yet to undergo rigorous controlled clinical trials to establish their efficacy [96]. Furthermore, unlike drugs, dietary supplements are not subject to FDA approval, resulting in an inadequate evaluation of their potential toxicities and interactions with other medications.

It has been reported that the oxidation of certain phenol ring-containing flavonoids by peroxidases can lead to cytotoxic phenoxyl radicals [99]. These radicals can co-oxidize several molecules, such as unsaturated lipids, NADH, ascorbate, and nucleic acids, producing ROS [100]. Reports show certain flavonoids are hepatotoxic, pro-oxidant, and have estrogenic activity [101]. Zhang et al. reported the potential toxicity of four common flavonoids (luteolin, apigenin, quercetin, and genistein), where concerns were raised about potential developmental toxicity, endocrine disruption, and mutagenicity [101].

Furthermore, various studies have reported the toxic effects of different flavonoids on various human organ systems, including the liver, kidney, gastrointestinal tract, skin, eyes, endocrine system, and brain [102]. Higher dosages of flavonoids may have mutagenic effects, promote oxidation by producing free radicals, and block crucial hormone metabolism enzymes. Because of this, care should be taken when taking flavonoids in amounts higher than those seen in a typical vegetarian diet. The potential harm to the developing baby should be of particular concern because flavonoids can cross the placental barrier. Given their rising usage, further studies must examine flavonoids’ toxicological characteristics [103].

The toxicity of flavonoids is influenced by several variables, including their type, dosage, and duration of usage. Based on only in vitro research, it is impossible to conclude the harmful effects of high dosages of flavonoids on humans. Although high flavonoid concentrations have been linked to genotoxicity in vitro experiments, food consumption rarely results in such amounts. Similarly, in vivo, tests have not consistently supported in vitro findings of quercetin-related mutagenicity and genotoxicity [103].

Additionally, the beneficial properties of flavonoids have been attributed to their antioxidant properties, and there is strong evidence showing that flavonoids can also have pro-oxidant properties, as mentioned previously [104]. For example, tea catechins, including EGCG, have demonstrated pro-oxidative effects in vitro, where it was observed that these catechins generated hydrogen peroxide [105]. Such prooxidative activities may have implications for potential toxicity. Moreover, rat hepatocytes treated with high concentrations of EGCG resulted in decreased cell viability. Some case reports about hepatotoxicity due to high doses of tea-based dietary supplements are also available [106,107]. Laboratory studies in animals have further supported the potentially toxic effects of high doses of green tea-derived preparations [108]. Additionally, some flavonoids have been reported to exhibit topoisomerase inhibitory effects with the potential to increase the risk of leukemia in offspring through the inhibition of topoisomerase II activity and subsequent chromosomal translocation [109,110].

A population-based association study in Japan reported that the consumption of isoflavones (genistein and daidzein) was associated with an increased risk of hepatocellular carcinoma (HCC) in women, and the association was dose-dependent [111]. Furthermore, there are some reports about flavonoid-related nephrotoxicity, such as *Taxus celebica* and Quercetin (one of the most commonly used flavonoids) [112,113,114]. 

## 5. Shortcomings of Flavonoids 

The bioavailability of flavonoids is a concern for producing therapeutic or health benefits. Flavonoids have low bioavailability due to low absorption, extensive metabolism and rapid excretion. Nevertheless, the different flavonoids have different degrees of bioavailability due to the structural variability of the flavonoid class. The discussed in vitro results have highlighted the potential of different flavonoids in many simulated disease conditions. Still, it is imperative to understand whether the concentrations used in these experiments are physiologically relevant. The same applies to animal studies where the administration of flavonoids to animals and pharmacokinetics must be rigorously evaluated. Dose concentrations in animal studies must also be physiologically relevant in different disease conditions. It is also essential to understand the metabolism of flavonoids and whether the metabolites reach the target organ, i.e., the brain. Because of intestinal absorption, flavonoids are rapidly metabolized, and it is imperative to look for metabolites in target organs and run in vitro cell-based experiments using metabolite concentrations rather than the parent compound. More comprehensive and rigorous studies in vitro and in vivo studies must be complemented by clinical studies to establish the natural flavonoid health benefits. 

## 6. Conclusions and Summary

As shown, flavonoids can exert their effects on many different biological pathways and have a strong therapeutic effect, mainly in antioxidants. While they are used in many different disease conditions, they also have shown a robust therapeutic impact on brain diseases and conditions. For example, when discussing neuroinflammation, certain flavonoids have shown that they can readily cross the BBB and modulate and control ROS levels. Lastly, they are relatively safe for consumption, as the sources of these flavonoids come from very commonly consumed food groups. While this information is promising, more studies regarding pharmacokinetics (absorption, distribution, metabolism and elimination) must be investigated to elucidate the bioavailability in brain regions further. In doing so, analogue structures of the flavonoid can be synthesized for potential neuro-therapeutic bioavailability. Currently, some flavonoids are in clinical phases for drug development; genistein, for example, is currently in clinical trials and seems to have promising results for anti-inflammatory diseases such as Alzheimer’s Disease. 

Future studies will elucidate the length and degree of flavonoid therapy and the primary and secondary mechanisms in which they may be involved. While dietary lifestyle changes may lead to small changes, isolating the active flavonoid component and developing a therapy may lead to more drastic and larger-scale modifications in terms of therapy. Regarding brain injury, flavonoids could be a safe and productive means of ameliorating the oxidative stress symptoms associated with brain injury, specifically strokes. Flavonoids have presented themselves as a strong candidate for therapies for brain diseases. As clinical trials proceed, the Food and Drug Administration will examine each one closely for the potential of new drug development. As the FDA approves more flavonoids for brain disorders, it is essential to understand which disorders they can treat and the degree to which they can help. For example, as stated, flavonoids’ therapeutic and anti-neuroinflammatory effects are worth investigating for treating certain diseases such as AD, PD, and strokes.

In conclusion, while flavonoids offer numerous benefits and have therapeutic potential for various neurological and neurodegenerative diseases, it is essential to consider their consumption’s potential adverse effects and toxicity. The review summarized the significance of the neurologic and neurodegenerative disease, the lack of specific therapeutic agents, and the use of dietary flavonoids devoid of side effects when taken in moderate amounts. We also discussed the neuropharmacology of flavonoids and possible side effects when taken in higher concentrations. However, further research is needed to understand better the safety profile and potential risks of flavonoid intake, particularly at high doses, long-term use, and in specific populations.
